# Accelerating Scientific Discovery Through Computation and Visualization

**DOI:** 10.6028/jres.105.068

**Published:** 2000-12-01

**Authors:** James S. Sims, John G. Hagedorn, Peter M. Ketcham, Steven G. Satterfield, Terence J. Griffin, William L. George, Howland A. Fowler, Barbara A. am Ende, Howard K. Hung, Robert B. Bohn, John E. Koontz, Nicos S. Martys, Charles E. Bouldin, James A. Warren, David L. Feder, Charles W. Clark, B. James Filla, Judith E. Devaney

**Affiliations:** National Institute of Standards and Technology, Gaithersburg, MD 20899-0001

**Keywords:** discovery science, distributed processing, immersive environments, IMPI, interoperable MPI, message passing interface, MPI, parallel processing, scientific visualization

## Abstract

The rate of scientific discovery can be accelerated through computation and visualization. This acceleration results from the synergy of expertise, computing tools, and hardware for enabling high-performance computation, information science, and visualization that is provided by a team of computation and visualization scientists collaborating in a peer-to-peer effort with the research scientists.

In the context of this discussion, *high performance* refers to capabilities beyond the current state of the art in desktop computing. To be effective in this arena, a team comprising a critical mass of talent, parallel computing techniques, visualization algorithms, advanced visualization hardware, and a recurring investment is required to stay beyond the desktop capabilities.

This article describes, through examples, how the Scientific Applications and Visualization Group (SAVG) at NIST has utilized high performance parallel computing and visualization to accelerate condensate modeling, (2) fluid flow in porous materials and in other complex geometries, (3) flows in suspensions, (4) x-ray absorption, (5) dielectric breakdown modeling, and (6) dendritic growth in alloys.

## 1. Introduction

Science advances through iterations of theory and experiment. Increasingly, computation and visualization are an integral part of this process. New discoveries obtained from an experiment or a computational model are enhanced and accelerated by the use of parallel computing techniques, visualization algorithms, and advanced visualization hardware.

A scientist who specializes in a field such as chemistry or physics is often not simultaneously an expert in computation or visualization. The Scientific Applications and Visualization Group (SAVG [1]) at NIST provides a framework of hardware, software and complementary expertise which the application scientist can use to facilitate meaningful discoveries.

Parallel computing allows a computer code to use the resources of multiple computers simultaneously. A variety of parallel techniques are available which can be used depending upon the needs of the application. Generally, parallel computing is thought of in terms of speeding up an application. While this is true, experience is showing that users often prefer to use this increased capability to do more computation within the same amount of time. This may mean more runs of the same complexity or runs with more complex models. For example, parallel computing can use the combined memory of multiple computers to solve larger problems than were previously possible. An example of this is described in Sec. 8, Dendritic Growth in Alloys.

Visualization of scientific data can provide an intuitive understanding of the phenomenon or data being studied. One way it contributes to theory validation is through demonstration of qualitative effects seen in experiments such as Jefferies orbits as described in Sec. 5, Flow of Suspensions. Proper visualization can also exhibit structure where no structure was previously known. In the Bose-Einstein condensate (BEC) example (Sec. 3), visualization was key to the discovery of a vortex array. Current visualization technology provides a full range of hardware and techniques from static two-dimensional plots, to interactive three-dimensional images projected onto a monitor, to large screen fully immersive systems allowing the user to interact on a human scale.

Immersive virtual reality (IVR) [2] is an emerging technique with the potential for handling the growing amount of data from large parallel computations or advanced data acquisitions. The IVR systems take advantage of human skills at pattern recognition by providing a more natural environment where a stereoscopic display improves depth perception and peripheral vision provides more context for human intuition.

The techniques used for parallel computing and visualization, as well as the knowledge of hardware, are specialized and outside the experience of most scientists. SAVG makes use of our experience in solving computational and visualization problems as we collaborate with scientists to enhance and interpret their data. Results of this work include theory validation, experiment validation, new analysis tools, new insights, standard reference codes and data, new parallel algorithms, and new visualization techniques.

## 2. Tools

SAVG has worked with many scientists at NIST on a wide variety of problems, and makes use of an array of resources that it can bring to bear on these diverse projects. Of course we make use of the central computing resources that include several SGI Origin 2000 systems,^1^ an IBM SP system, a cluster of PCs running Linux, as well as virtual parallel machines created from workstation clusters. Each of these systems can be used for parallel as well as sequential implementations of scientific algorithms. In addition to these central computing resources, SAVG uses commercial tools and freely available tools where appropriate, augmenting these with locally developed tools when necessary. The following are some tools in common use by SAVG.

### 2.1 Computation

#### MPI—Message Passing Interface

The majority of our parallel applications are written using the message-passing model for parallel programs. In the message-passing model each process has exclusive access to some amount of local memory and only indirect access to the rest of the memory. Any process that needs data that is not in its local memory obtains that data through calls to message passing routines. MPI is a specification for a library of these message-passing routines. Since its introduction in 1994, MPI has become the de facto standard for message-passing programming and is well supported on high performance machines as well as on clusters of workstations and PCs.

MPI was designed to support its direct use by applications programmers as well as to support the development of parallel programming libraries. We have used MPI in both of these contexts (see the descriptions of C-DParLib, F-DParLib, and AutoMap/AutoLink below).

Interoperable MPI (IMPI) [6, 7] is a cross-implementation communication protocol for MPI that greatly facilitates heterogeneous computing. IMPI enables the use of two or more parallel machines, regardless of architecture or operating system, as a single multiprocessor machine for running any MPI program. SAVG was instrumental in the development of the IMPI protocol.

#### C-DParLib and F-DParLib

The libraries C-DParLib and F-DParLib [8, 9, 10, 11],, developed by SAVG, support data-parallel style programming in C and Fortran 90, respectively. These libraries make heavy use of MPI to handle all communication. Data-parallel programming refers to parallel programming in which operations on entire arrays are supported such as A = A + B, where A and B are arrays of values. C-DParLib and F-DParLib were developed specifically to support parallel applications that derive parallelism primarily from the distribution of large arrays among processing nodes such as in most finite-difference based parallel applications.

Both libraries support basic data-parallel operations such as array initialization, array shifting, and the exchanging of array data between adjacent processing nodes. In addition, C-DParLib provides additional services such as assisting in the balancing of computational loads among the processing nodes and the generation of arrays of pseudo-random values. Both libraries are portable to any platform that supports MPI and C or Fortran 90.

#### OpenMP

A standardized, portable tool set for implementing parallel programs on shared-memory systems in C, C++, and Fortran [12, 13].

#### AutoMap/AutoLink

Tools for simplifying the use of complex dynamic data structures in MPI-based parallel programs [14]. This software was developed at NIST and is fully portable to any platform that supports MPI and C [15, 16, 17, 18].

#### WebSubmit

A Web-based interface [19] that simplifies remote submission of jobs to NIST’s heterogeneous collection of high-performance computing resources. It presents a single seamless user interface to these diverse platforms. WebSubmit was developed at NIST and is portable to other environments [20, 21].

### 2.2 Visualization

#### OpenDX—Open Data Explorer

An open-source visualization software package [22] with a sophisticated data model. OpenDX can take advantage of multiple processors on a high performance multiple CPU system. OpenDX is very useful for the rendering of volumetric data.

#### IDL—Interactive Data Language

A commercially available high-level language [23] used for data processing and analysis. Many standard analysis routines such as image processing are included as easily callable functions. Additionally, IDL has routines for developing graphical user interfaces (GUI) allowing rapid development of powerful interactive two and three dimensional graphics.

#### Interactive Graphics Workstations

SAVG maintains a Visualization Laboratory where high performance graphics workstations are made available for collaborators. These workstations provide a facility for NIST scientists to run a wide range of interactive computational and visualization software.

#### OpenGL—Performer

A commercial product for performance-oriented 3D graphics applications. Performer [24] provides a scene graph API (application programming interface) and the ability to read a variety of data formats.

#### RAVE—reconfigurable automatic virtual environment

A commercially available product which provides a visually immersive environment for data display and interaction. It is driven by an SGI Onyx2 visual supercomputer. Our current configuration has a single 2.29 m×2.44 m rear projection screen utilizing Crystal Eyes active stereoscopic glasses with head and wand tracking.

#### DIVERSE

The primary software library used to develop RAVE applications. Developed at the Virginia Tech CAVE, DIVERSE [25] software has the advantage of providing a device-independent virtual environment. The same application can run on a desktop workstation as well as on single and multi-wall immersive systems. In addition, the software is based on SGI’s OpenGL Performer allowing applications to take advantage of a wide variety of Performer data formats. These design features can provide an application continuum from the desktop to the visualization lab to the RAVE.

#### VRML—Virtual Reality Modeling Language

A Web based standard that allows interactive viewing of three dimensional data. SAVG uses VRML [26] as a mechanism for distributing visualizations and graphical simulations to collaborators.

#### Non-linear Video Editing

A computer/disk based video editing system that allows random access to video and computer graphics sources. Because it is digital, sophisticated editing techniques such as motion effects, keying, titling, and resizing can easily be used. Also, it is very easy to create movies in many different digital formats for dissemination over the Internet, or movies can be written out in several video tape formats for use at presentations and meetings or for distribution.

The computation and visualization resources described here, together with the expertise to use them, enable SAVG to collaborate on a wide range of research problems with NIST scientists.

## 3. Bose-Einstein Condensates

A Bose-Einstein condensate (BEC) is a state of matter that exists at extremely low temperatures. BECs were first predicted in 1925 by Albert Einstein as a consequence of quantum statistics [27].

### 3.1 Scientific Background

Researchers at the National Institute of Standards and Technology are studying BECs of alkali atoms confined within magnetic traps. These studies are conducted through numerical simulation as well as laboratory experiments. Numerical simulation of BECs is addressed by solving the appropriate many-particle wave equation. The wave function of a BEC corresponds to a macroscopic quantum object. In other words, a collection of atoms in a BEC behaves as a single quantum entity and is therefore described by a single wave function.

**Fig. 1:**
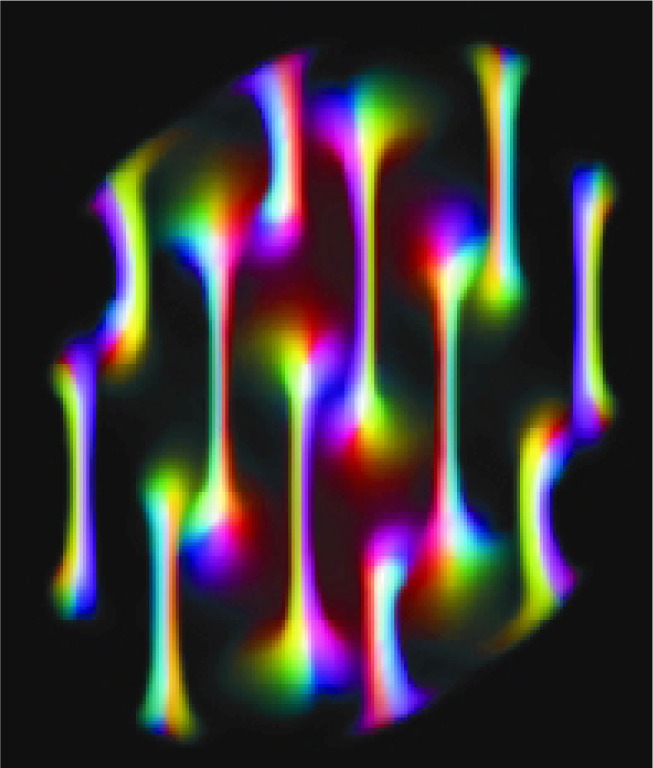
Array of vortices in a Bose-Einstein condensate under rotation.

The evolution of the BEC wave function is in question when the trapped BEC is subjected to rotation. Upon rotation, quantized vortices may form within the BEC. These vortices are of interest because of their theoretical implications for the characteristics of BECs, such as superfluidity (see Fig. 1).

Researchers perform numerical simulations of the BEC wave function based on first principles to determine if quantized vortices exist in these systems. A typical result of such a simulation is a sequence of three-dimensional arrays of complex numbers. Each complex number reflects the value of the BEC wave function at a particular position and time.

### 3.2 Data Analysis

Simulations of rotating BECs are computed on a three-dimensional grid of order 100 grid points along each dimension. The simulation data are subsequently interpolated onto a mesh with 200 points in each dimension for the purposes of visualization. When each complex number is decomposed into two real components, there are 16×10^6^ scalar values to consider at each time step. Traditional line and surface plots, for example, are not adequate for the investigation of three-dimensional qualitative features such as vortices. More suitable techniques, such as scientific visualization, are required.

### 3.3 Visualization

In some respects, scientific visualization is a generalization of traditional two-dimensional plotting and graphing. One goal of visualization is the creation of a single “picture” that conveys to the researcher a small number of high-level concepts. A collection of such pictures may be concatenated into an animated sequence to convey concepts that vary over position and time, for example.

In the case of BECs, the goal of the visualization task is to identify and isolate possible vortex structures within a three-dimensional volume. Volume rendering techniques are appropriate for this situation. In particular, the volume rendering model used for this investigation assumes that each point in three-dimensional space both emits and absorbs light.

In an abstract sense, the visualization of a three-dimensional array of BEC data requires the construction of a function to map from the BEC data domain to an image domain. The BEC data domain is composed of three-dimensional positions along with complex values from the associated wave function. The image domain consists of an opacity component and three color components: hue, saturation, and brightness. Opacity describes the extent to which a point in three-dimensional space absorbs light. Hue describes the gradation among red, green, or blue. Saturation describes the degree of pastelness. Brightness describes the degree of luminance.

The construction of a function from the BEC data domain to the image domain proceeds as follows: The complex values associated with the wave function are decomposed into polar form. The angular component of a complex value determines the hue by mapping the angle to a corresponding position on a color circle. A color circle, as used here, begins with red at 0 radians and then traverses through green and blue with a return to red at the completion of the circular trip. The radial component of a complex value determines the brightness by mapping small radii to high brightness and large radii to low brightness. The radial component of the brightness mapping corresponds to density, where low density regions are bright. The intent is to exhibit low-density vortices as bright regions and suppress the visibility of high-density regions. The saturation is determined by a constant function; all regions are fully saturated. Finally, the opacity is determined by a constant function as well; all regions have zero opacity (that is, complete transparency).

The function described above is further modified with respect to the magnetic trap in which the BEC exists. The purpose of this modification is the suppression of unimportant regions beyond the confines of the magnetic trap. The BEC in the magnetic trap is ellipsoidal in shape and the required modifications are straightforward applications of analytic geometry.

### 3.4 Results

**Fig. 2:**
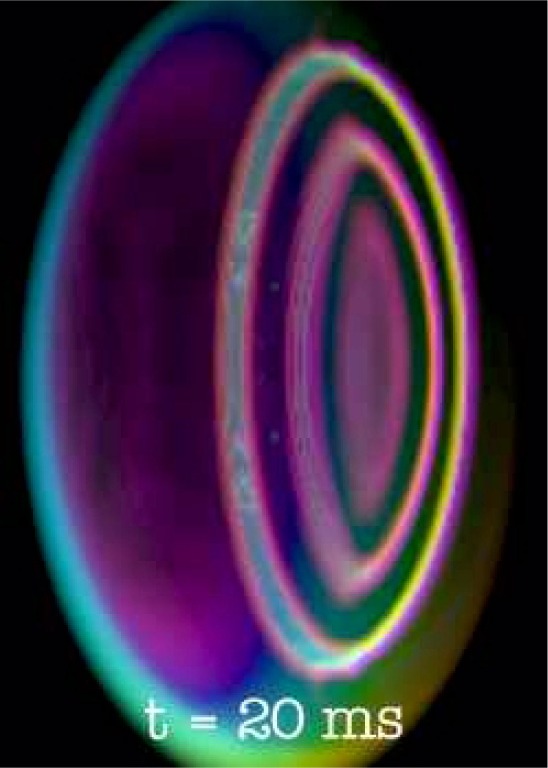
Soliton produced by phase imprinting of a Bose-Einstein condensate.

The result of the visualization process is a sequence of images, one for each time step, which form a 3D stereoscopic animation. In this study, the BEC images did indeed show the presence of quantized vortices. In addition, the visualization also discovered an unanticipated structure of concentric vortex rings, shown in Fig. 2, instead of the line vortices as shown in Fig. 1. Further, the images are the first three-dimensional visualization of vortex structures in a rotating BEC [28].

Additionally, a BEC image of a soliton, produced at the trap center by a phase imprinting technique, looks like a flat disk, corresponding to a low-density plane within the condensate cloud. As the soliton propagates through the condensate, it becomes more curved because the soliton moves fastest in the condensate center, and doesn’t move at all at the condensate surface. At a later time, the entire soliton stops completely and becomes a nodal surface. Rather than returning to the point of creation, it spontaneously decays into concentric quantized vortex rings, in a process known as a *snake instability*; see Fig. 2 [29].

This instability provoked a great deal of further simulations and calculations. The results were presented in Ref. [30].

Experimentalists at JILA, Brian Anderson and Eric Cornell, attempted to generate these vortex rings in condensates in exactly this way. They have confirmed all the predictions.

## 4. Fluid Flow in Porous Materials and in Other Complex Geometries

The flow of fluids in complex geometries plays an important role in many environmental and technological processes. Examples include oil recovery, the spread of hazardous wastes in soils, and the service life of building materials. Further, such processes depend on the degree of saturation of the porous medium. The detailed simulation of such transport phenomena, subject to varying environmental conditions or saturation, is a great challenge because of the difficulty of modeling fluid flow in random pore geometries and the proper accounting of the interfacial boundary conditions.

**Fig. 3:**
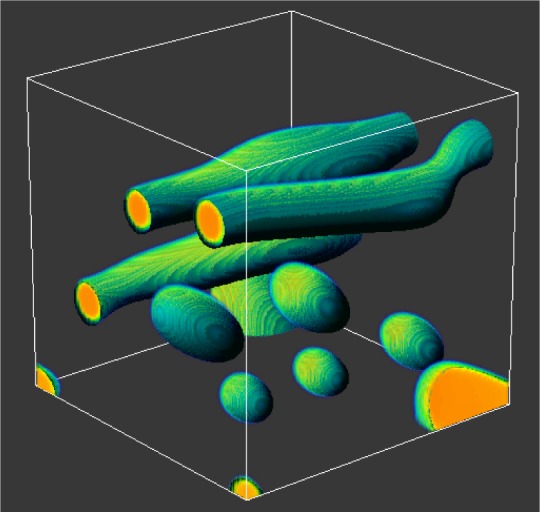
Phase separating binary mixture under shear simulated using a lattice Boltzmann method.

The work described here involves the application of the lattice Boltzmann (LB) method to this problem. The LB method of modeling fluid dynamics naturally accommodates multiple fluid components and a variety of boundary conditions such as the pressure drop across the interface between two fluids and wetting effects at a fluid-solid interface. Indeed, the LB method can be applied to a wide variety of complex flow problems that strongly depend on boundary conditions including phase separation of polymer blends under shear, flow in microchannel devices, and the modeling of hydrodynamic dispersion. For example, Fig. 3 shows an LB simulation of a phase separating binary mixture under shear [31]. The LB and related methods are currently in a state of evolution as the models become better understood and corrected for various deficiencies [32, 33].

One difficulty with LB methods is that they are resource intensive. In general, running simulations on large systems (greater than 100^3^ grid points) is not practical due to the lack of memory resources and long processing times. Because of these extreme demands on memory and computation, and the fact that the LB method generally needs only nearest neighbor information, the algorithm was an ideal candidate to take advantage of parallel computing resources.

### 4.1 Implementation of the LB Algorithm

The approach of the LB method is to consider a typical volume element of fluid to be composed of a collection of particles that are represented by a particle velocity distribution function for each fluid component at each grid point. The time is counted in discrete time steps and the fluid particles can collide with each other as they move, possibly under applied forces.

The sequential implementation of the algorithm was relatively straightforward. We have both active sites (that hold fluid) and inactive sites (that consist of material such as sandstone). For efficient use of memory we use an indirect addressing approach where the active sites point to fluid data and the inactive sites point to NULL. Hence only minimal memory needs to be devoted to inactive sites. At each active site we point to the necessary velocity and mass data for each fluid component. Over the course of an iteration we visit each active cell in the data volume and calculate the distribution of each fluid component to be streamed to neighboring cells. New mass and velocity values are accumulated at each active cell as its neighbors make their contributions.

We implemented the parallel version of the algorithm using the Message Passing Interface [3] (MPI). The parallelization was accomplished within a simple single-program multiple-data (SPMD) model. The data volume is divided into spatially contiguous blocks along the *z* axis; multiple copies of the same program run simultaneously, each operating on its own block of data. Each copy of the program runs as an independent process and typically each process runs on its own processor. At the end of each iteration, data for the planes that lie on the boundaries between blocks are passed between the appropriate processes and the iteration is completed.

The mechanisms for exchanging data between processes via MPI calls and for managing the minor housekeeping associated with MPI are concealed within a few routines. This enables us to have a purely serial version of the program and a parallel version of the code that are nearly identical. The code is written in standard ANSI C, and the only external library that has to be used is the MPI library, which is available on all of NIST’s parallel systems as well as many other parallel computing environments. These implementation strategies enable us to run the program, without any modification on any of NIST’s diverse computing platforms.

### 4.2 Verification

**Fig. 4:**
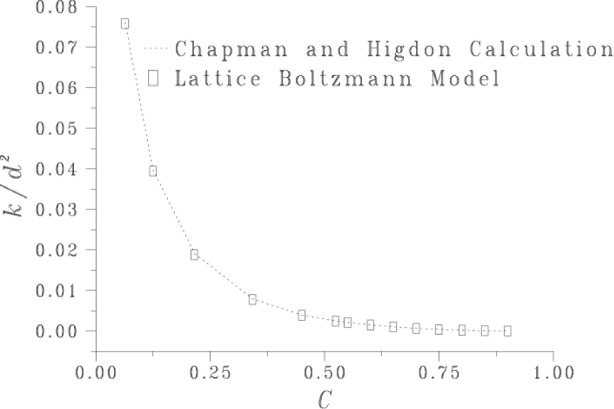
Normalized flow through spheres, as a function of the solid fraction *C*, centered on a simple cubic lattice. The permeability *k* is normalized by the square of the distance *d* between sphere centers. The solid fraction *C* is (1—porosity).

We verified the correctness of the model with several numerical tests. For example, one test involved computing the permeability of porous media composed of a periodic array of (possibly overlapping) spheres. In Fig. 4 we compare our simulation data with those of Chapman and Higdon [34], which are based on the numerical solution of coefficients of a harmonic expansion that satisfies the Stokes equations. Agreement is very good, especially given that the solid inclusions are digitized spheres.

**Fig. 5:**
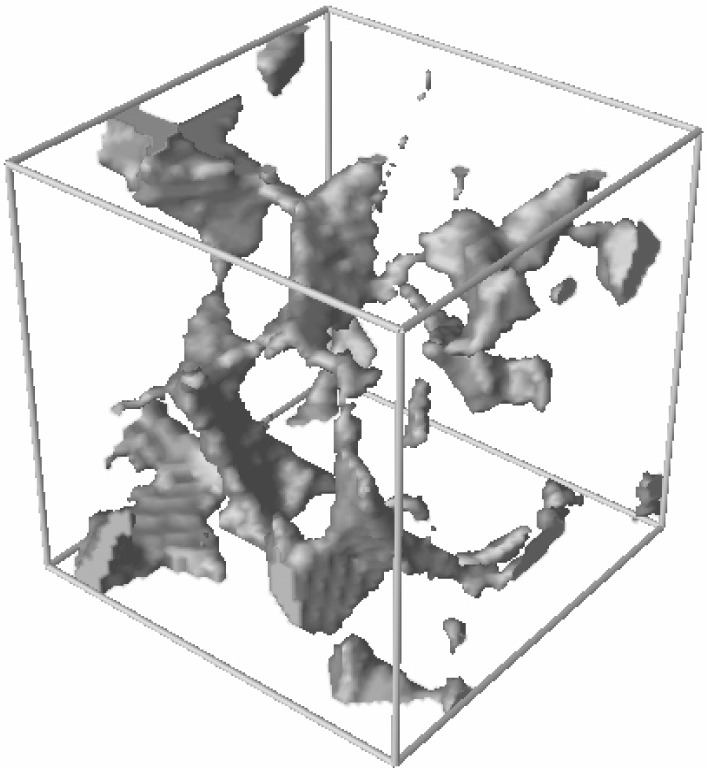
A 64^3^ portion of the 7.5 % porosity Fontainebleau sandstone media. The solid matrix is made transparent to reveal the pore space (grey shaded region).

**Fig. 6:**
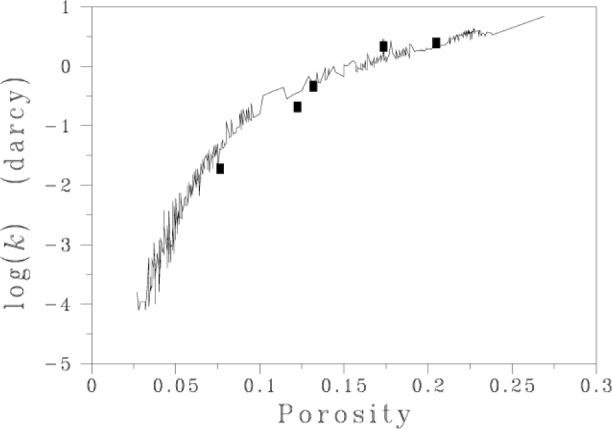
Measured and modeled permeabilities (*k*) of Fontainebleau sandstone media as a function of porosity. The solid rectangles show the modeled results.

We then determined the permeability of several microtomography-based images of Fontainebleau sandstone. Figure 5 depicts a portion of one of these sandstone images. The resolution is 5.72 μm per lattice spacing and data sets were 510^3^ voxels (volume elements). Figure 6 shows the computed permeability compared to experimental data [35]. Clearly there is good agreement, especially at the higher porosities.

### 4.3 Performance of the Parallel Code

We ran a series of timing tests on several of the parallel systems at NIST, including an SGI Origin 2000, an IBM SP2, and an Intel Pentium cluster. Because of the portability of the MPI calls and our standard ANSI C code it was easy to run the same code and test cases on each platform.

The timings recorded for these runs closely agree with a very simple model describing performance:

T=S+P/N,

where

*T* is total time for a single iteration,
*S* is time for the non-parallelizable computation,
*P* is time for the parallelizable computation, and
*N* is number of processors.

The parallelizable computation is that portion of the processing that can be effectively distributed across the processors. The non-parallelizable computation includes processing that cannot be distributed; this includes time for inter-process communication as well as computation that must be performed either on a single processor, or must be done identically on all processors.

We found in all cases that the non-parallelizable computation *S* accounts for between 0.7 % and 3 % of the total computational load. In one of the test cases the performance data from the SGI Origin 2000 closely matches this formula (*T* is the total time in seconds for an iteration):

T=0.090s+11.98s/N.

The non-parallizable computation *S* is 0.090 s, while the parallelizable portion of the computation *P* uses 11.98 s. So, for example, a single iteration took 12.08 s on one processor but only 1.11 s on 12 processors. These results indicate that the algorithm is, indeed, well suited to a parallel computing environment.

Other timing tests indicate that the time for the parallelizable portion of the code is roughly proportional to the number of active sites over the entire volume, while interprocess communication time is roughly proportional to the size of an *xy* cross-section of the volume. So as we process larger systems, the time for the parallelizable portion of the code should increase proportionally with the cube of the linear size of the system, while the non-parallelizable portion should increase with the square of the linear size of the system. This means that for larger systems, a larger proportion of the time is in the parallelizable computation and greater benefits can be derived from running on multiple processors.

### 4.4 Results

The modeled permeabilities of the Fontainebleau sandstone media and their agreement with experimental results verified the correctness and utility of our parallel implementation of the LB methods. These simulations would not have been possible without parallelizing the algorithm. The requirements for computing resources are beyond the capacity of single-processor systems.

In addition, parallelization has enabled us to try alternatives that would have been prohibitive in the past. For example, when calculating the permeabilities of the Fontainebleau sandstone samples, we found that at the lowest porosity (7.5 %), there were not enough nodes across the pores to produce a reliable flow field. Because we could handle large volumes, we were able to double the resolution on a large subset of the low-porosity sample. This yielded very satisfactory results, as indicated above.

Lattice Boltzmann methods for simulating fluid flow in complex geometries have developed rapidly in recent years. The LB method produces accurate flows and can accommodate a variety of boundary conditions associated with fluid-fluid and fluid-solid interactions. With the advent of parallel systems with large memories, computations on large systems that were considered beyond the reach of even some “super” computers from a few years ago can now be considered routine.

## 5. Computational Modeling of the Flow of Suspensions

Understanding the flow properties of complex fluids like suspensions (e.g., colloids, ceramic slurries, and concrete) is of technological importance and presents a significant theoretical challenge. The computational modeling of such systems is also a great challenge because it is difficult to track boundaries between different fluid/fluid and fluid/solid phases. Recently, a new computational method called dissipative particle dynamics (DPD) [36] has been introduced which has several advantages over traditional computational dynamics methods while naturally accommodating such boundary conditions. In structure, a DPD algorithm looks much like molecular dynamics (MD) where particles move according to Newton’s law. That is, in each time step, the forces on each particle are computed. The particles are then moved and the forces recalculated. However, in DPD, the interparticle interactions are chosen to allow for much larger time steps so that physical behavior, on time scales many orders of magnitude greater than that possible with MD, may be studied. The original DPD algorithm used an Euler algorithm for updating the positions of the free particles (which represent “lumps” of fluid), and a leap frog algorithm for updating the positions of the solid inclusions. Our algorithm QDPD [37] is a modification of DPD that uses a velocity Verlet [38] algorithm to update the positions of both the free particles and the solid inclusions. In addition, the solid inclusion motion is determined from the quaternion-based scheme of Omelayan [39] (hence the Q in QDPD).

QDPD uses an implementation of the linked cell method [40, 41] which is a true *O*(*N*) algorithm. The QDPD cell is partitioned into a number of subcells. For every time step a linked list of all the particles contained in each subcell is constructed. The selection of all pairs of *particles* within the cutoff is achieved by looping over all pairs of *subcells* within the cutoff and particles within the subcells. Because of their regular arrangement, the list of neighboring subcells is fixed and may be precomputed.

QDPD was originally written in Fortran 77 as a serial program. To improve performance, a parallelization of the code was done in MPI [42] using a simplified version of the replicated data approach.

### 5.1 Replicated Data Approach

In the replicated data approach [43, 44, 45] every processor has a complete copy of all the arrays containing dynamical variables for every particle. The computation of forces is distributed over processors on the basis of cell indices. This is a very efficient way of implementing parallelism since the forces must be summed over processors only once per time step, thus minimizing interprocessor communication costs. On “shared-memory” machines like an SGI Origin 2000, this approach is very attractive, since all processors can share the arrays containing dynamical variables.

The biggest disadvantage of the replicated data strategy is that every processor must maintain a copy of all of the data and therefore the data must be updated on each processor at the end of each time step. This is not a problem in the shared-memory multiprocessor version if the MPI implementation is smart enough to take advantage of the shared memory. In our implementation, a global sum technique is used to add the separate contributions to the forces via an MPI_Allreduce library call. This approach has worked well for small to medium sized problems (tens-of-thousands of particles) on the shared-memory SGIs. We have found speedups of as much as 17.5 times on 24 processors of a 32 processor SGI Origin 2000. Utilizing three such systems, we were able to complete a year’s worth of conventional computing in a week. Among the results obtained by this technique has been the calculation and subsequent visualization of a sheared suspension of ellipsoids.

### 5.2 Spatial Decomposition

While the replicated data approach of the previous section has been the workhorse of QDPD work for some time now, it has had its disadvantages. The biggest disadvantage is that scaling to very large numbers of processors in a shared-memory environment is poor (24 is the practical limit for us), and it has turned out to be almost unusable on distributed memory systems including those with high speed interconnects like the IBM SP2/SP3 systems.

When the goal is to simulate an extremely large system on a distributed-memory computer to allow for the larger total memory of the distributed-memory computer and also to take advantage of a larger number of processors, a different approach is needed. Our spatial decomposition [46, 47] replaces the serial linked cell algorithm with a parallel linked cell algorithm [44, 48]. The basic idea is this:

Split the total volume into *P* volumes, where *P* is the number of processors. If we choose a one dimensional (1D) decomposition (“slices of bread”), then the *p*th processor is responsible for particles whose *x*-coordinates lie in the range

$$(p-1)M_{x}/P\leq x\leq pM_{x}/P,$$

where *M_x_* is the size of the volume along the *x* axis.

Similar equations apply for 2D and 3D decompositions for simulation dimensions *M_y_* and *M_z_*. Whether the decomposition is 1D, 2D, or 3D depends on the number of processors: First assign particles to processors. Augment particles on each processor with neighboring particles so each processor has the particles it needs. Now on each processor, form a linked cell list of all particles in the original volume plus an extended volume that encompasses all of the particles that are needed for computations on this processor. Loop over the particles in the original volume, calculating the forces on them and their pair particle (for conservation of momentum). Care must be taken to add these forces on particles in the extended volume to the forces on the processor “owning” them. Finally calculate the new positions of all particles and move the particles which have left the processor to their new home processors.

We distinguish between “owned” atoms and “other” atoms, where the later are atoms that are on neighboring processors and are part of the extended volume on any given processor. For “other” atoms only the information needed to calculate forces is communicated to neighboring processors. Second, the QDPD technique is being applied to suspensions, so there are two types of particles: “free” particles and particles belonging to solid inclusions such as ellipsoids. A novel feature of this work is that we explicitly do *not* keep all particles belonging to the same solid inclusion on the same processor. Since the largest solid inclusion that might be built can consist of as many as 50 % of all particles, it would be difficult if not impossible to handle in this way without serious load-balancing implications. What we do is assign each particle a unique particle number when it is read in. Each processor has the list of solid inclusion definitions consisting of lists of particles defined by these unique particle numbers. Each processor computes solid inclusion properties for each particle it “owns,” and these properties are globally summed over all processors so that all processors have solid inclusion properties. Since there are only a small number of solid inclusions (relative to the number of particles), the amount of communication necessary for the global sums is small and the amount of extra memory is also relatively small. Hence it is an effective technique.

Current results show a speed up of a factor of 22.5 on 27 200 MHz Power3 processors on an IBM SP2/SP3 distributed memory system. The same technique also is very effective in a shared-memory environment, where the speedups are a factor of 29 on 32 processors of an SGI Origin 3000 system and a factor of 50 on 64 processors.

### 5.3 Visualization

While various quantitative tests are used to help validate our algorithms, visualization plays an important role in the testing and validation of codes. Even simple visual checks to make sure the solid inclusions satisfy boundary conditions can be helpful.

**Fig. 7:**
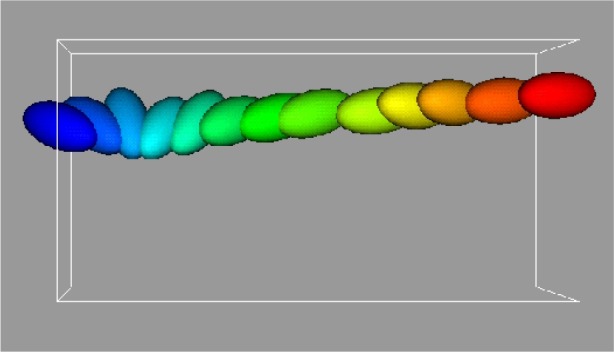
Motion of a single ellipsoidal inclusion subject to shear. The single ellipsoid rotation is a well known phenomenon seen in experiments called Jefferies orbits.

Figure 7 shows a time series of the motion of a single ellipsoidal inclusion subject to shear. The different colors correspond to the time sequence. The shearing boundary conditions were obtained by applying a constant strain rate to the right at the top of the figure and to the left at the bottom. Note that the single ellipsoid rotates. This is a well known phenomenon seen in experiments called Jefferies orbits.

**Fig. 8:**
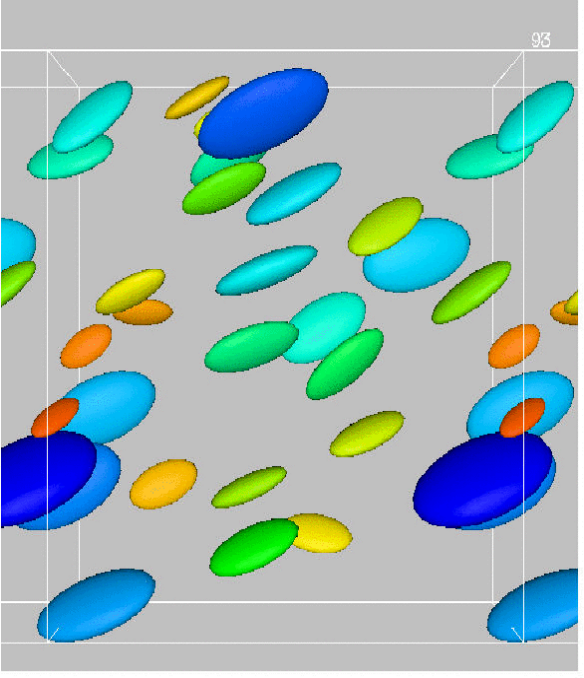
Motion of twenty eight ellipsoidal inclusions, of size varying up to a factor of two, subject to shear. Note that the Jefferies orbits are suppressed due to hydrodynamic interactions between ellipsoids.

In contrast, we found that when several elliposidial inclusions were added to the system (Fig. 8) the Jefferies orbits were suppressed and the ellipsoids had a tendency to align as their relative motion was partly stabilized by mutual hydrodynamic interactions.

**Fig. 9:**
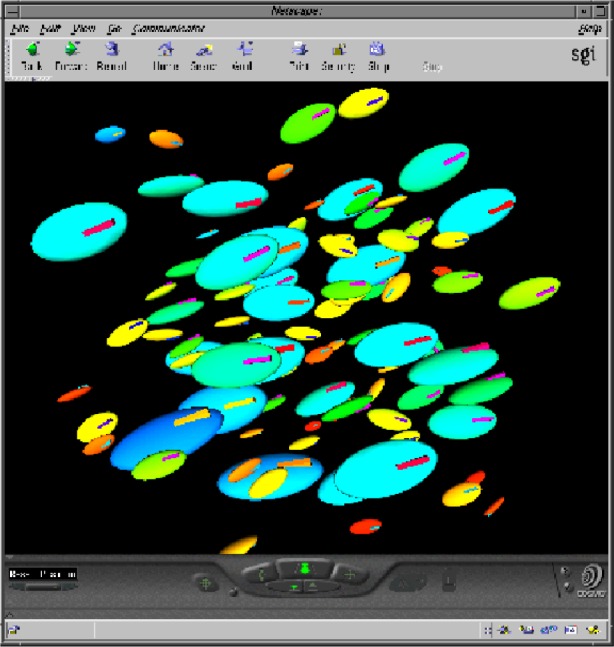
A screen shot of a Web based animation using VRML to allow interactive viewing of the time series animation.

Virtual Reality Modeling Language (VRML) [26] has been used to distribute animations of the results from this computation (Fig. 9). VRML is a Web-based standard that allows interactive viewing of three dimensional data. In contrast to movie loop animations, VRML allows the user to interactively view the animation while the results of the computational model is cycled. This interactive viewing capability allows users to select their own point of view. Since it is Web based, the animation can be distributed to any PC or UNIX based system with a VRML viewer installed. The amount of data displayed and speed of viewing is only limited by the speed of the viewing system. An example of using VRML to animate the results from a computational model of the flow of suspensions can be found on the Web [49].

## 6. Rapid Computation of X-Ray Absorption Using Parallel Computation: FeffMPI

X-ray absorption spectroscopy (XAS) uses energy-dependent modulations of photoelectron scattering to determine local atomic structure [50]. XAS is usually divided into the extended x-ray absorption fine structure (EXAFS) with photoelectron energies above approximately 70 eV, and the x-ray absorption near edge structure (XANES) in the 0 eV to 70 eV range. Theoretical calculations of photoelectron scattering are now an integral part of both EXAFS and XANES analysis. These theoretical calculations have grown in sophistication and complexity over the past 20 years. Fortunately, during the same time period, Moore’s law [51] has increased computing power dramatically, so that EXAFS calculations are now fast, accurate, and easily executed on inexpensive desktop computers [52, 53]. However, XANES calculations remain time-consuming in spite of these improvements. The photoelectron mean free path is large at the low photoelectron energies of the XANES region, so accurate XANES calculations require large atomic clusters and remain challenging on even the fastest single processor machines. Furthermore, the photoelectron scattering is strong for low energies, so that full multiple scattering calculations are required. These calculations require repeated inversions of large matrices which scale as the cube of the size of the atomic cluster [54]. Further sophistication in the computer codes, such as the use of non-spherically symmetric potentials, will improve accuracy but increase computational requirements even further. The computation required for XANES calculations led us to investigate the use of parallel processing.

To implement parallel processing of XANES we started from the serial version of the computer code Feff [54]. Feff (for effective potential *F*_eff_) does real-space calculations of x-ray absorption, is written in portable Fortran 77, and uses a number of computational strategies for efficient calculations. Our goal was to implement a parallel processing version of Feff that retained all the advantages and portability of the single-processor code while gaining at least an order of magnitude improvement in speed. Feff models the physical process of x-ray absorption, so it was natural to exploit the intrinsic task or *physical* parallelism, namely, that x-ray absorption at a given x-ray energy is independent of the absorption at other energies. We use this physical parallelism to make simultaneous calculations of the XANES at different energies using multiple processor clusters, and then assemble the results from the individual processors to produce the full XANES spectrum. We use the Message Passing Interface (MPI) to implement this idea [42]. We have run the parallel Feff code (FeffMPI) on Linux, Windows NT, IBM-AIX, and SGI systems with no changes to the code. FeffMPI can run on any parallel processing cluster that supports MPI, and these systems can use distributed or shared memory, or even a mixture of distributed and shared memory.

The starting point for “parallelizing” Feff was to determine which parts of the code were the most time consuming. As expected on physical grounds, profiling tests showed that the loop over x-ray energies in the XANES computation dominated the time; over 97 % of the CPU time is spent inside this loop. Therefore, we chose this part of the code for the initial work on a parallel version of Feff. A secondary hot spot is a similar loop that is used to construct self-consistent potentials. In this first version of FeffMPI the self-consistency calculation does not execute in parallel; we plan to implement this in a later revision.

By concentrating on a single hot spot in the code, we leave 99.7 % of the existing single-processor code of Feff unchanged. We use the MPI libraries to arbitrarily designate cluster node number one as the *master* node, and designate the other *N*_procs_–1 nodes as *workers*. In the energy loop of the XANES calculation each node (*master* and *workers*) executes 1/*N*_procs_ XANES calculations that each cover 1/*N*_procs_ of the energy range of the XANES calculation. After each *worker* completes its part of the task, the results are sent back to the *master* and the *worker* processes can be terminated. This approach means that (1) exactly the same executable is run on every node in the cluster; (2) virtually all of the changes to the single-processor Feff are confined within a single subroutine; (3) the FeffMPI code is nearly identical to the single-processor version of Feff, the only difference being that each instance of the FeffMPI process is aware that it is a particular node of a cluster of *N*_procs_ processors; and (4) communication between *master* and *worker* processors is kept to a minimum.

**Fig. 10:**
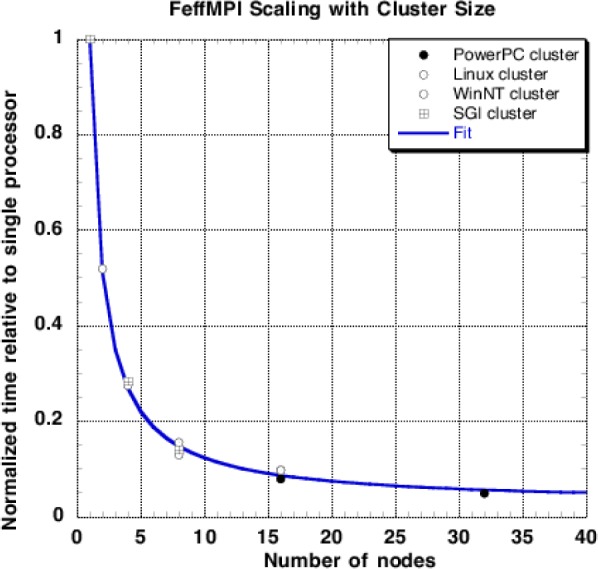
Runtime of a typical FeffMPI XANES calculation with cluster size. The calculation has been run on four different clusters. The execution time on a single processor has been normalized to 1.0, showing that the scaling on all clusters is very similar once the variation in processor speed and compiler quality is eliminated. The scaling indicates that about 3 % of the runtime is still from the sequentially executing parts of the code, implying that a very large cluster should run FeffMPI about 30 times faster than an equivalent single processor.

To evaluate how well the parallel algorithm succeeds, we conducted tests on six systems. As representative single-processor systems, we did benchmarks on a 450 MHz AMD K6-3 running SuSe Linux 6.1, and an Apple PowerMac G4 running at 450 MHz. We then ran FeffMPI on four MPI clusters: (1) a cluster of 16 Pentium II 333 MHz systems running Redhat Linux connected via 100 Mbit Ethernet; (2) a similar cluster of Pentium III 400 MHz machines running Windows NT connected by 100 Mbit Ethernet; (3) a cluster of SGI machines; and (4) an IBM SP2/3 using up to 32 processors. The fastest times were turned in by using 32 IBM SP3 processors. That system was 25 times faster than the PowerMac G4 and 40 times faster than the single processor Linux system. We found that processing speed could be predicted, as a function of cluster size, by the simple scaling law *T* = *α*(0.03 s + 0.97 s/*N*_proc_), where *T* is the runtime in seconds (s), α is a scaling factor that accounts for the speed of a given single processor type and the efficiency of the compiler, and *N*_proc_ is the number of processors in the cluster. As shown in Fig. 10, if the runtimes on the various clusters are rescaled by the α for that cluster, giving a normalized runtime of 1.0 for each cluster when a single processor is used, all the runtimes fall on a universal curve that shows how well FeffMPI scales with cluster size. As cluster size is increased, the part of the code that runs in parallel changes from the dominant part of the runtime to an irrelevant fraction of the total. In the limit of large cluster sizes, runtime is dominated by the 3 % of the original code that still executes sequentially. In such large clusters, we expect no further increase in speed because the runtime is then totally dominated by sequentially executing code. In fact, large clusters can even *increase* runtime due to communications overhead. However, on the largest clusters we had available, we did not observe any saturation of the scaling due to communication overhead.

### 6.1 Results on Parallel Processing Clusters

As one example of these calculations, we show how XANES measurements are used in the study of barium-strontium titanate (BST) films that are of interest as high-k dielectrics in electronic devices [55, 56]. The films are deposited by metal-organic chemical vapor deposition (MOCVD) that must take place at low substrate temperatures because of processing constraints in device fabrication. Due to the low deposition temperature the structure of the films often departs from the ideal crystalline BST state [57]. However, the actual structure is unknown and the structural origin of the variation in the dielectric constant is undetermined. Because the films contain amorphous material that gives no clear x-ray diffraction signal, we used XANES measurements to help understand the structure of the films and *ab initio* calculations using FeffMPI to interpret the XANES spectra.

**Fig. 11:**
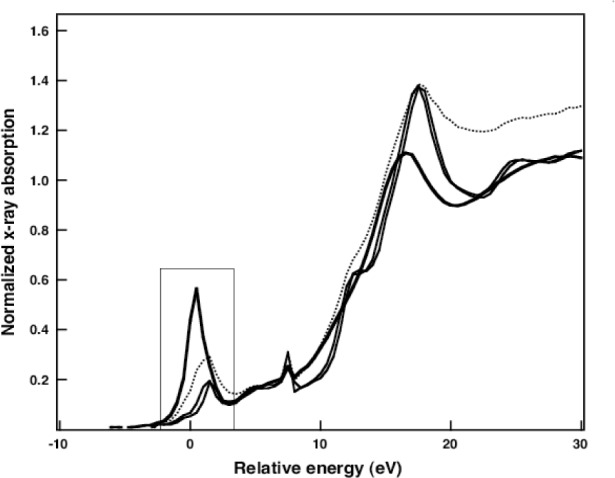
Measured XANES data of 4 Barium-strontium titanate (BST) films deposited by MOCVD. The variation in size and energy position of the pre-edge peak near −2 eV to +2 eV is a signature of the structural variation in these films.

**Fig. 12:**
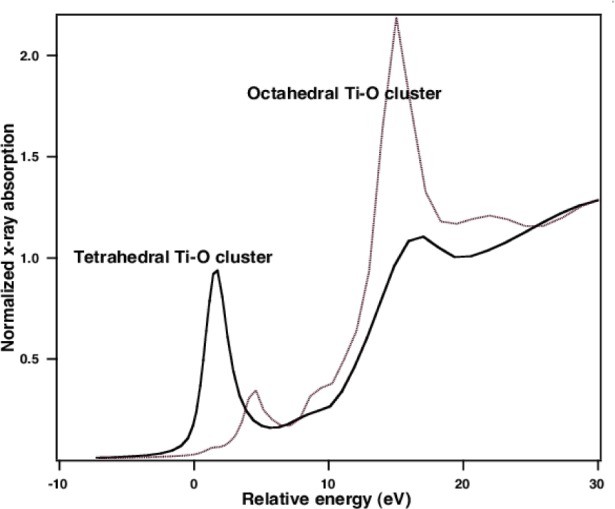
XANES calculation from the octahdral and tetrahedral Ti-O structures shown in Figs. 13 and 14. The nearly perfect inversion symmetry of the Ti-O octahedra leads to only a small low-energy resonance in the XANES. The non-inversion symmetric tetrahedral Ti-O environment gives a much larger low-energy resonance. The qualitative similarity of these simulations with the XANES measurements shown in Fig. 11 indicates that the BST films make a transition from a non-ferroelectric phase with tetrahedral Ti-O oxygen coordination to the octahedral Ti-O structure that is characteristic of Ba-TiO_3_.

In Fig. 11 we show a series of XANES measurements of several BST films. The most important feature is the evolution of the peak near −2 eV to +2 eV (the origin of the energy zero is arbitrary) as deposition conditions are changed. In Fig. 12 we show theoretical calculations of tetrahedral and octahedral oxygen coordination around the Ti atoms; note the qualitative similarity to the trend seen in the measured XANES data in Fig. 11.

**Fig. 13:**
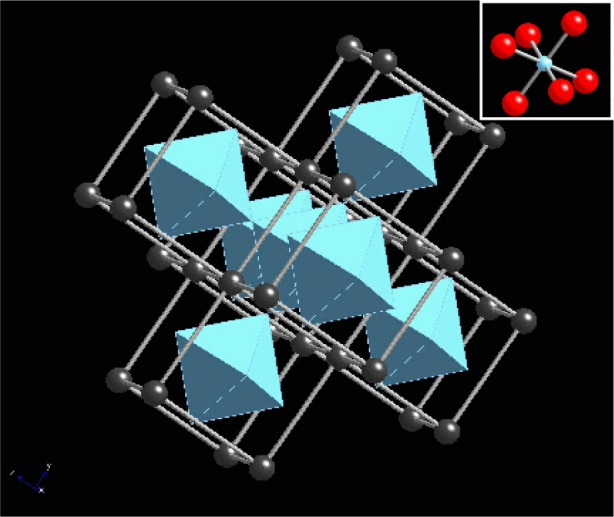
Rendering of the ideal rhombohedral structure of BaTiO_3_. The structure is a repetition of nearly perfect Ti-O octahedra that are separated by a nearly cubic cage of Ba atoms. The nearly perfect inversion symmetry of the Ti-O octahedra leads to only a small low-energy resonance in the XANES. Except for a mixture of both Ba and Sr atoms on the same site, this is the expected structure for BST films deposited with high substrate temperatures.

**Fig. 14:**
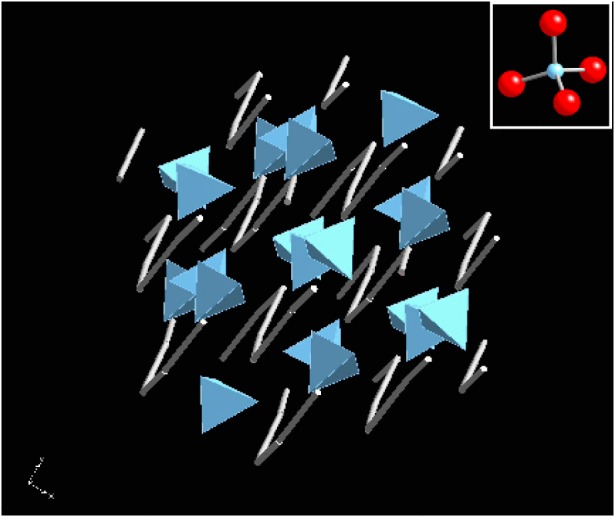
Rendering of the structure of Ba_2_TiO_4_. The structure is a repetition of nearly perfect Ti-O tetrahedra that are rotated with respect to each other and are separated by zig-zag chains of Ba atoms. The lack of inversion symmetry in the Ti-O tetrahedra leads to a very large low-energy resonance in the XANES.

The calculations suggest that the observed change in the XANES implies a change from a non-inversion symmetric Ti-O structure with tetrahedral oxygen coordination to one that is a nearly inversion symmetric octahedral Ti-O arrangement. The tetrahedral Ti-O structures are not ferroelectric, so this structural variation accounts for the change of the dielectric constant with film deposition temperature and titanium-oxygen stoichiometry. In Figs. 13 and 14 we show the structures of BaTiO_3_ and Ba_2_TiO_4_ that were used as the inputs for the calculations in Fig. 12. The Ba_2_TiO_4_ structure has a slightly distorted Ti-O tetrahedral structure with zig-zag chains of Ba atoms separating the Ti-O tetrahedra. The BaTiO_3_ structure contains Ti-O octahedra with nearly perfect inversion symmetry, and the octahedra are surrounded by a cage of Ba atoms. The BST films contain amorphous material which are probably distortions of those shown in Figs. 13 and 14, but we can say with certainty that the Ti-O environment changes from one with inversion symmetry to one that is strongly non-inversion symmetric. Chemical constraints and the FeffMPI calculations suggest that this is because of a transition from octahedral to tetrahedral oxygen coordination.

## 7. Dielectric Breakdown Modeling; Growth of Streamers in Dielectric Liquids

In high-voltage power transformers, catastrophic breakdown in the dielectric oil is preceded by the rapid growth of conducting plasma streamers. Branching filamentary structures sometimes form in the streamers, as documented through high-speed photographic experiments conducted by Hebner, Kelley, and Stricklett at NBS in the 1980s [58]. However, the photographs did not record the very fast processes (on the order of tens or hundreds of nanoseconds) that caused the filament to develop. Our model describes the “shaping” effects of the surrounding electric field on the rapidly-growing plasma streamers.

We have applied stochastic Laplacian growth as a model to filamentary dielectric breakdown as described by Pietronero and Wiesmann [59] and others [60, 61, 62, 63]. Here we construct a simplified model of the algorithm on a large Cartesian grid using boundary conditions which confine the electric field. We examined the effect of parameters (threshold voltage, choice of power law) on the fractal structure (which can be dense or sparse) and the timing of the growth process. The calculation of the voltage field throughout the full volume, which is repeated after each iteration of breakdown growth, is the major computational burden. The computational resources required for this problem suggested the use of parallel methods.

### 7.1 Implementation

Our first parallel implementation of the algorithm was developed in a machine-language which was specific to the CM-2 Connection Machine. This version of the code used a single instruction, multiple data (SIMD) model which fits our problem closely. The current parallel method was then developed in a portable serial version using the array-oriented features of Fortran 90. The Fortran 90 array operations and intrinsic functions enabled us to write the code in a very compact form that closely corresponds to the mathematical description of the underlying algorithm. Furthermore, these features of Fortran 90 greatly simplified the parallelization of the code.

The serial code was converted to parallel by using our F-DParLib subroutine library. F-DParLib is designed to be used in a single-program-multiple-data (SPMD) programming approach. In other words, multiple copies of the same program are running simultaneously, and each copy is processing a different portion of the data. In particular, F-DParLib provides simple mechanisms to divide very large arrays into blocks, each of which is handled by a separate copy of the program. In practice, this means that the researcher can write parallel code that looks almost identical to serial code. In our case, the code could be written as though addressed to a single active grid-node and its immediate neighbors. Fortran 90, extended across block boundaries by F-DParLib, executed each instruction on all sites of each array.

F-DParLib’s emphasis on array handling is designed to mesh with Fortran 90’s array syntax and intrinsic array-handling functions. Much of F-DParLib consists of parallel versions of the intrinsic array functions such as CSHIFT and MAXVAL.

In parallelizing this code, F-DParLib played the role of a high-level language for block parallelism. Using F-DParLib we converted the existing serial version of the algorithm to a parallel version with very few changes. The parallel version of the code can easily be run, without modification, on many processors on a large parallel system, or on a single processor on a desktop workstation.

Multiple parallel algorithms were implemented to speed the runs. Spatial decomposition through block decomposition required each processor to track only its part of the space. Parallel breakdown was also implemented using a randomized red-black algorithm. Laplace’s equation was solved in parallel using SOR [64]. Finally, time compression was used to reduce the empty (no breakdown) steps for periods of low breakdown probability [65].

### 7.2 Results

The parallel computing model was validated by comparison of model visualization to photographs taken of streamers during physical experiments [66, 65, 67]. These images enable us to make a detailed, qualitative comparison of features of the model versus those of the actual phenomenon being modeled. We have also used animation and color banding of the images to simulate time progression.

**Fig. 15:**
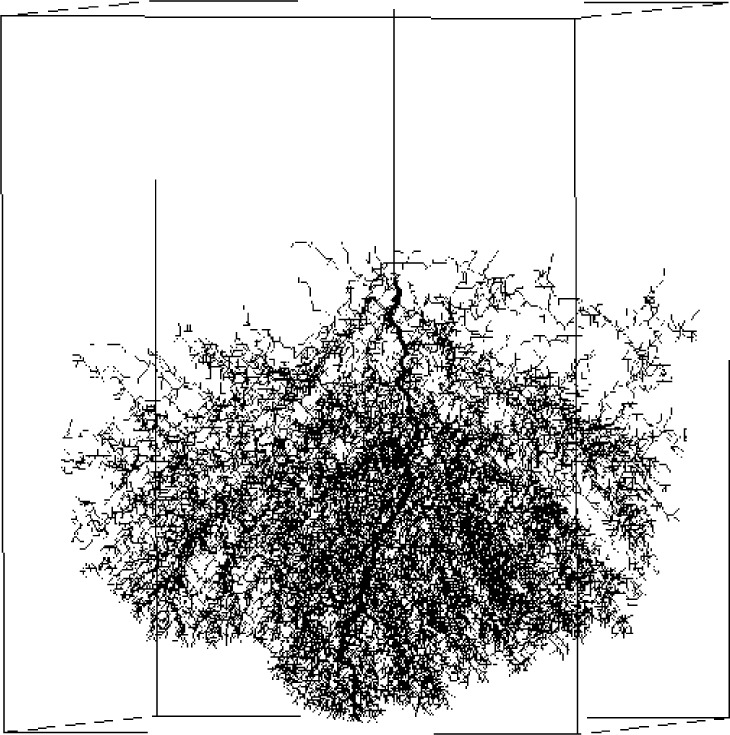
Simulation of a dense streamer growth associated with a low cutoff-voltage parameter.

**Fig. 16:**
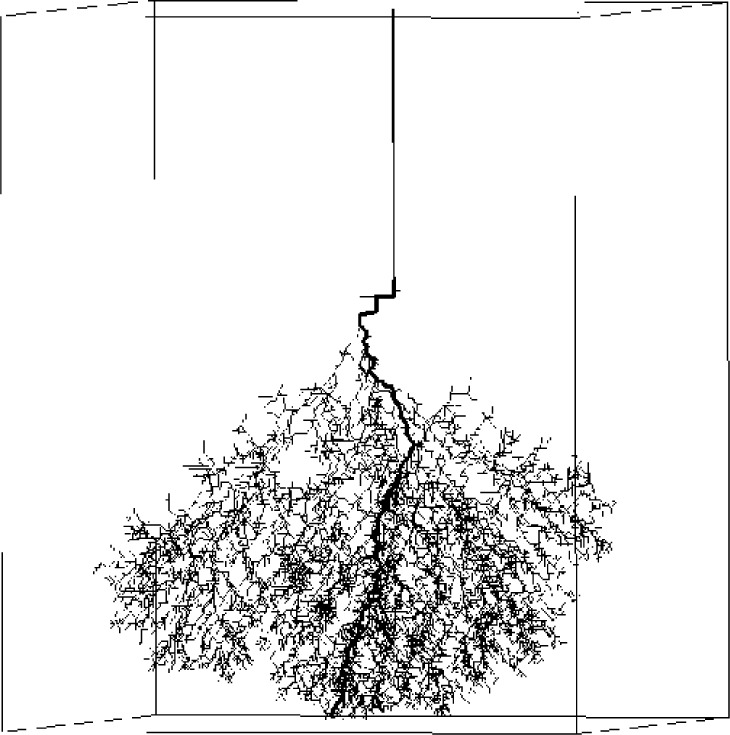
The conical top envelope of the streamer is narrowed by increasing the cutoff-voltage parameter. The narrowing has a counterpart in experimental behavior under increased pressure.

Our algorithm has reached a new level of detail and realism for this class of simulations. The trend from sparse, forward-directed growth to volume-filling side-branching has been illustrated for a range of power-law response curves. Several parallel algorithms have been included in the numerical modeling. We have simulated a range of effects which occur in experiments as the parameters of the model are changed. For example, Figs. 15 and 16 demonstrate a narrowing of the conical top envelope associated with increased cutoff voltage, which has its experimental counterpart in experimental behavior under increased pressure.

## 8. Dendritic Growth in Alloys

### 8.1 Background

When an alloy is cast, the liquid metal freezes into a solid in much the same way that water freezes to form ice. Just as water freezes forming intricate patterns called snowflakes, cast alloys also form snowflake-like patterns. These treelike structures are generically known as dendrites, and are ubiquitous in microstructural casting patterns.

A better understanding of the process of dendritic growth during the solidification of alloys is the goal of this project. This knowledge will help guide the design of new alloys and the casting process used to produce them.

Early versions of computational models of solidification, known as sharp interface models, treated the liquid-solid interface as a mathematically two-dimensional boundary. Tracking this complicated boundary was a computationally challenging task [68, 69, 70].

In the phase field method, however, the liquid-solid transition is described by an order parameter that determines, at each location in the simulated alloy, whether the alloy is in the liquid or solid phase. The transition from liquid to solid is not abrupt, but follows a smooth curve, thus allowing the interface to have thickness and internal structure. The phase field method can determine the exact location and movement of the surface of the dendrite during the simulation by simply updating each point in the phase-field on each time step of the iteration according to the relevant governing equations. This algorithm, in two-dimensions, is described in detail by Warren and Boettinger [71].

Our collaboration on this project began when the researchers wished to expand their two-dimensional simulation to three dimensions. The new simulation would better match the actual three-dimensional nature of these dendrites, as well as verify physical properties of dendrites that only appear when all three dimensions are included. The amount of computing power as well as the amount of memory needed to simulate this process of dendrite growth in three dimensions required more hardware than was available on the desktop.

### 8.2 Implementation

Our three-dimensional simulation of dendritic growth is of a copper-nickel alloy. A pair of diffusion equations, one describing the phase-field and one describing the relative concentration of the two solutes, is solved over a uniform three-dimensional grid using a first-order finite difference approximation in time and second-order finite difference approximation in space. On each time-step of this algorithm, each point in the grid is updated. At regular intervals, a snapshot of the phase-field and concentration are saved to disk for later processing into animations and still pictures of the simulated dendrite.

A three-dimensional grid of size 1000×1000×1000 is required to obtain the detailed and highly resolved images needed from this simulation. Eight three-dimensional arrays of this size are required, each containing double precision (8 byte) floating point elements. Therefore, this program requires over 64 GB of memory for the desired resolution. In order to handle such a large amount of data, we have developed a parallel version of this simulator.

We have used MPI [3, 4] to develop a data-parallel style program that can be run efficiently on both distributed memory and shared memory machines. The MPI-based communication library C-DParLib [8, 9] has been used to simplify the coding of this simulator. Sufficient parallelism is obtained by distributing the three-dimensional arrays among the available processors and exchanging data between adjacent processors at the beginning of each time step. Currently, the arrays are distributed along one axis but they could be distributed along two or three axes if needed.

Parallel applications benefit when the computational load on each processor is approximately the same. Given a homogeneous set of processors, load-balancing sometimes can be accomplished simply by distributing the elements of the arrays equally among the processors. Unfortunately, this balancing is only effective if the processors are identical and the computational load is the same at all points throughout the finite-difference grid. Nether of these assumptions are true for this simulator. The update algorithm requires more computations at grid points near the surface and inside the dendrite compared to the rest of the grid, so distributing the arrays equally, even assuming perfectly equal processors, results in a load imbalance. In modern computing facilities, such as at NIST, as parallel machines are upgraded, an originally homogeneous set of processors commonly becomes heterogeneous over time with the introduction of higher speed processors and processing nodes with local memories of varying sizes. This effect has resulted in heterogeneous parallel machines at NIST.

At run time, our C-DParLib [72] can periodically redistribute the arrays across the processors according to simple performance parameters, such as execution time per element, for each iteration. This can greatly improve the execution time depending on the set of processors that are assigned to the job. The impact of this dynamic load-balancing on the source code for the simulator is small with only a few C-DParLib function calls required within the main iteration loop.

### 8.3 Visualization

**Fig. 17:**
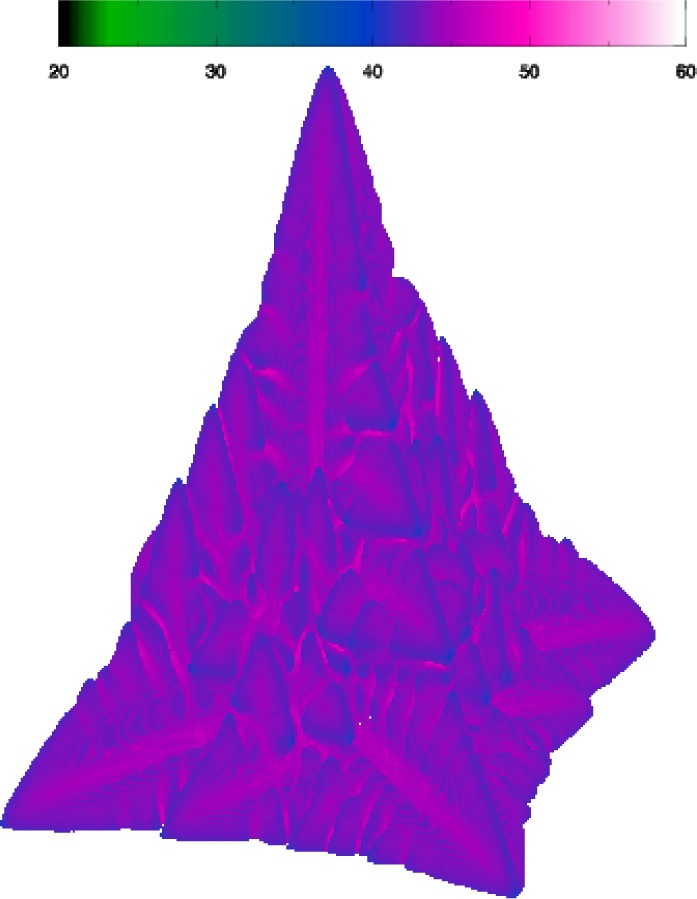
A 3D dendrite from a simulation over a grid of 300^3^ points. The color bar shows the coding of the relative concentration of the metals in the dendrite. The color coding ranges from concentrations of 20 % to 60 %.

The output from this simulator consists of pairs of files (snapshots) containing the phase-field (*ϕ*) and the relative concentration (*C*) of the solutes at each grid point at specific time steps. For each simulation, we produce 40 snapshots at regular time intervals. TIFF (Tagged Image File Format) images are made from the snapshot data, then replayed sequentially to generate an animation showing the dendrite as it grows. At the smaller grid sizes, below 300^3^, we use commonly available visualization software to process these snapshot files into color images with appropriate lighting and shading added to enhance the images. In this process, we interpret the value of 128 (mid-point of the byte-scaled data) in the phase field to be the surface of the dendrite and calculate an isosurface of the phase-field data using this value. The surface is then colored using the relative concentration of the alloys from the data in the corresponding *C* snapshot file. An example of this for a simulation on a grid of size 300^3^ is shown in Fig. 17.

**Fig. 18:**
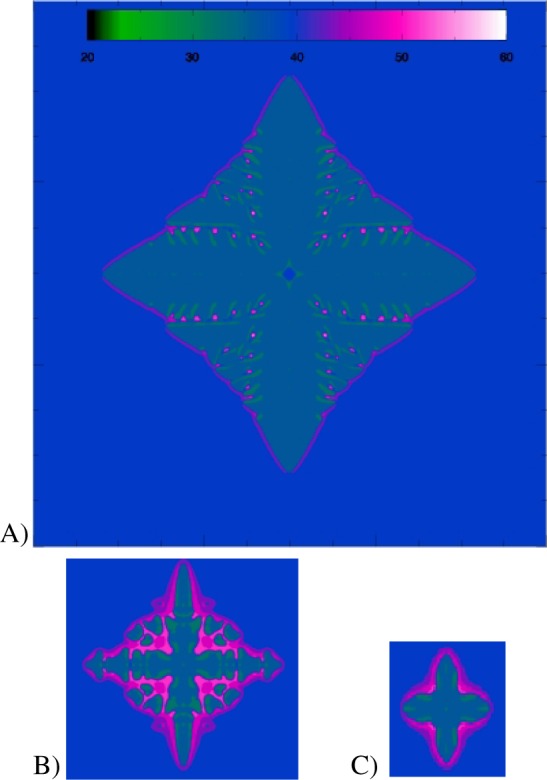
Three 2D slices through the 3D dendrite shown in Fig. 17. The scale is the same in these three images but in order to save space the area surrounding the dendrite has been clipped. The color coding used in these images is identical to the color coding used in Fig. 17. The blue background corresponds to the initial concentration of approximately 40 %. Image A is a slice through the base of the dendrite, image B is a slice taken halfway down toward the tip of dendrite, and image C is a slice taken near the tip of the dendrite.

Two-dimensional slices through these snapshots are also produced to investigate the details of the internal structure. Three slices through the dendrite shown in Fig. 17 are shown in Fig. 18. Animations of both the dendrite and slices through the dendrite are generated.

Simulations on grids of size 300^3^ and larger cannot use this technique due to the increased memory requirements in calculating the isosurface. Although our machines have the available main memory to complete an isosurface calculation on these larger grids, most software is not capable of utilizing all of the available memory due to addressing limitations (32 bit limits). In addition to this addressing limitation, the commonly available visualization systems do not provide interactive viewing in a 3D movie loop of the dendrite growth. We have therefore begun to investigate alternative methods for visualizing these snapshots.

**Fig. 19:**
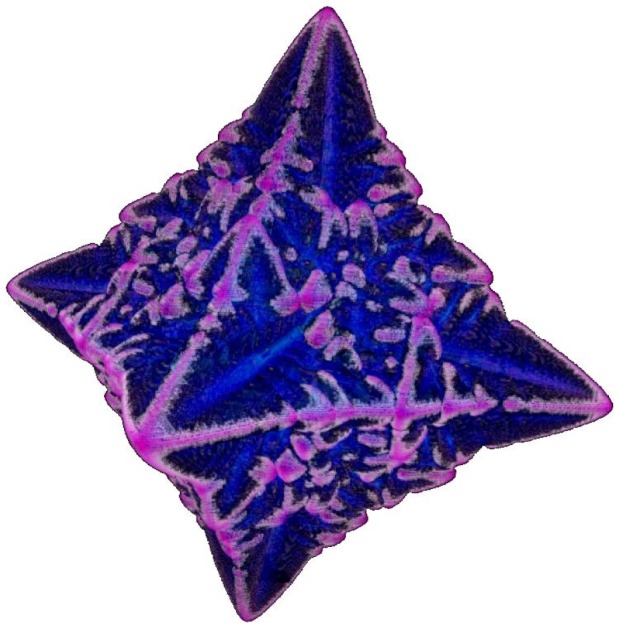
A 3D dendrite visualized using glyphs and semi-transparent colors. This image was generated from the same data as in Fig. 17. In this image the output from the simulator has been mirrored along all three axes giving a symmetric six-pointed star structure. The image in Fig. 17, due to memory limitations in computing the isosurface, was mirrored only along the *x* and *y* axes.

The SGI Onyx2 systems have high performance hardware that can provide interactive viewing for large amounts of polygonal data. We have developed a visualization procedure that converts the 3D grid data into a polygonal data set that can take advantage of this hardware acceleration. Each data point within the dendrite, i.e., with a phase of 128 or less, is represented by a glyph of three planar quadrilaterals oriented in each of the three orthogonal planes (*xy, xz, yz*). The size of these glyphs correspond to the 3D grid voxel size. A semitransparent color value as a function of concentration is assigned to the glyph. A full color scale ranging from black to white represents low to high areas of concentration. The speed of the interactive display is determined by the number of glyphs (polygons) used to form the dendrite. As previously stated, phase values in the range of 0 to 128 are inside the dendrite. Interactivity can be increased by restricting the range of the values selected for glyphs. For example, Fig. 19 uses glyphs for phase values from 28 to 128. However, the trade off for increasing interactivity is a more sparse representation of the dendrite. Using standard SGI software, OpenGL Performer, this polygonal representation is easily displayed. The semi-transparent colors allow a certain amount of internal structure to be revealed and the additive effects of the semi-transparent colors produces an isosurface approximation. A series of polygonal representations from the simulator snapshots are cycled producing a 3D animation of dendrite growth that can be interactively viewed. Most of the currently available immersive virtual reality (IVR) systems are based on OpenGL Performer. Thus, utilizing this format immediately allows the dendrite growth animation to be placed in an IVR environment for enhanced insight.

### 8.4 Status

Our largest three-dimensional dendritic growth simulation to date has been on a grid of size 500^3^ using 32 processors of an IBM SP. This simulation took approximately 70 h to complete. With the increase in the number and speed of available processors on our systems, and the associated additional memory, we will be able to regularly run simulations on grids of size 1000^3^.

Test runs on our current systems, which include an IBM SP, a Linux based PC cluster, several SGI Origin 2000 machines, and other available SGI workstations, indicate that we will soon be able to complete a 1000^3^ simulation in 3 to 4 days. This assumes that we can run on 70 to 80 of these compute nodes, and that each includes 1 GB of main memory or more. At that point we will begin production runs of this simulator. The use of IMPI (Interoperable MPI) [6] is expected to assist us in utilizing the compute nodes from these different machines as a single large heterogeneous parallel machine.

The 3D phase-field simulator enabled by parallel computing will provide a better understanding of the solidification phenomena and increased understanding of the parameter space as it pertains to melting. Dendritic growth models are an important element of macroscale commercial solidification packages, which will be the the eventual users of our results.

## 9. Conclusion

To maintain our ability to provide world class computational support for our scientific collaborations, we expect that NIST will continue to upgrade its central computing facility with current generation high-performance parallel computation servers as well as clusters of high performance PCs. Beyond this, SAVG will continue to develop and apply advanced parallel scientific computing and visualization techniques that enable us to run the largest, most detailed, and most useful computational experiments possible.

The newly installed Reconfigurable Automatic Virtual Environment (RAVE) is the next step for SAVG to improve our acceleration of scientific discovery. This system provides a large rear projection screen for peripheral vision, stereoscopic display for increased depth perception, and head tracking for more realistic perspective. All of the features combine into an immersive environment for greater insight into the collaborative results.

Our collaborations free physical scientists to focus on their science and the output of these computational experiments while we focus on the raw computational and visualization problems. The goal in these efforts is always to advance the scientific research of our collaborators.

## Footnotes

1 Certain commercial equipment, instruments, or materials are identified in this paper to foster understanding. Such identification does not imply recommendation or endorsement by the National Institute of Standards and Technology, nor does it imply that the materials or equipment identified are necessarily the best available for the purpose.
